# Playground safety assessment by Safety Report Card (SRC) method in Qom, Iran

**Published:** 2019-08

**Authors:** Alireza Koohpaei, Faezeh Javadi, Mohsen Sadeghi yarandi, Alireza Mashkoori, Ali Ebrahimi, Mohammad Khandan

**Affiliations:** ^*a*^Occupational Health Department, Health Faculty, Qom University of Medical Sciences, Qom, Iran.

## Abstract

**Background:**

Play is an important part of each child s life and is classified among the first children activities. One of the important issues that have been little attention to it, is a safety of playgrounds. Without any doubt the safety of children requires a safe environment and safe equipment that will use in playgrounds. This study aiming at playground safety assessment in Qom city by Safety Report Card (SRC) method in 2017-2018 was conducted.

**Methods:**

In this cross-sectional study, 98 park has been evaluated in Qom city. In order to evaluation the safety of playground, after assessing the validity and reliability of Safety Report Card method, mentioned method was used. Data were analyzed by using Independent sample t-test, correlation coefficient, Analysis of variance (ANOVA), Chi-Square statistical tests with SPSS software version 20. Finally, with regard to safety levels of playgrounds, GIS maps for the playgrounds safety in the Qom City were drawn.

**Results:**

The results of this study showed that the mean scores of the parks studied in SRC method is a low and equal to 12.18. The highest average scores were related to repair and maintenance of play equipment and the lowest mean scores were also related to the fall in danger. Results related to the frequency of each of the safety levels of SRC method indicated that 3.1% of the playgrounds were at the “F” safety level, 52% of the playgrounds were at the “D” safety level, 43.9% of the playgrounds were at the “C” safety level and 1% of the playgrounds were at the “B” safety level. Also there was a meaningful relationship between the parts of the fall in danger in the SRC method with the level of incomes in various urban areas.

**Conclusions:**

The result has shown that the majority of playgrounds in Qom city has a low safety level. Considering that playgrounds play an important role in the growth and development of children s personality and also the vitality and dynamics of their cities, corrective actions in order to making safe playgrounds and sustainable development of the city, is recommended.

**Keywords:**

Safety, Playground, Safety Report Card (SRC), Qom City

